# Scaling Behavior of Human Locomotor Activity Amplitude: Association with Bipolar Disorder

**DOI:** 10.1371/journal.pone.0020650

**Published:** 2011-05-31

**Authors:** Premananda Indic, Paola Salvatore, Carlo Maggini, Stefano Ghidini, Gabriella Ferraro, Ross J. Baldessarini, Greg Murray

**Affiliations:** 1 Department of Neurology, University of Massachusetts Medical School, Worcester, Massachusetts, United States of America; 2 Department of Psychiatry, Harvard Medical School, Boston, Massachusetts, United States of America; 3 International Consortium for Psychotic and Bipolar Disorders Research, McLean Hospital, Belmont, Massachusetts, United States of America; 4 Section of Psychiatry, Department of Neuroscience, University of Parma, Parma, Italy; 5 Forensic Hospital, Mental Health Department of Reggio Emilia, Reggio Emilia, Italy; 6 Faculty of Life and Social Sciences, Swinburne University of Technology, Hawthorn, Victoria, Australia; University of Maribor, Slovenia

## Abstract

Scale invariance is a feature of complex biological systems, and abnormality of multi-scale behaviour may serve as an indicator of pathology. The hypothalamic suprachiasmatic nucleus (SCN) is a major node in central neural networks responsible for regulating multi-scale behaviour in measures of human locomotor activity. SCN also is implicated in the pathophysiology of bipolar disorder (BD) or manic-depressive illness, a severe, episodic disorder of mood, cognition and behaviour. Here, we investigated scaling behaviour in actigraphically recorded human motility data for potential indicators of BD, particularly its manic phase. A proposed index of scaling behaviour (*Vulnerability Index* [VI]) derived from such data distinguished between: [i] healthy subjects at high versus low risk of mood disorders; [ii] currently clinically stable BD patients versus matched controls; and [iii] among clinical states in BD patients.

## Introduction

*Scale invariance* is a feature of biological complexity arising from interactions of various physiological control nodes operating at multiple time scales [1]. Loss of scale invariance can indicate pathophysiological states as it represents a shift to fewer control nodes [2]. In humans, the hypothalamic suprachiasmatic nucleus (SCN) is a major node in neural networks responsible for multi-scale regulation of biological rhythms. In particular, the SCN is responsible for scale-invariant properties of temporal fluctuations in locomotor activity [3]. Multi-scale properties of activity data can be important clinically, and are readily detected non-invasively and recorded by wrist-worn, microprocessor-controlled, piezoelectric actigraphic devices. Multi-scale features of physiological data have proved useful in understanding various clinical states as diverse as heart disease [4], [5], asthma [6], schizophrenia [7], and clinical depression [7], [8], [9].

Bipolar (manic-depressive) disorder (BD) is a relapsing, major psychiatric disorder with poorly understood neurobiology, typically incomplete treatment responses, and often unsatisfactory clinical outcomes, with a high risk of premature mortality due to suicide and other causes. BD may be particularly well suited to the investigation of multi-scale properties of motility data. Its complex manifestations include marked disturbances of locomotor activity [10], [11] as well as major changes in mood, thinking, and many aspects of behavior. Furthermore, the SCN may be involved in the regulation of behavior and mood in BD patients [12]. Development of non-invasive, objective correlates or predictors of morbid states in BD patients may support more timely and efficient clinical interventions aimed at limiting risk of recurrence of major episodes of illness.

Based on this background, we sought biologically plausible correlates of BD in multi-scale characteristics of daily motility rhythms in human subjects, including patients with reliably diagnosed BD in a range of ill and remitted clinical states, healthy controls, and subjects showing a range of potential vulnerabilities for BD based on standardized and validated behavioural and symptomatic ratings. We hypothesized that multi-scale behavior in motility rhythms recorded over several days would: [1] distinguish between high and low vulnerability to BD in apparently healthy young adults, [2] distinguish BD patients from healthy, sex- and age-matched controls, and [3] distinguish among different psychopathological states of BD patients.

We tested these hypotheses using data collected with wrist-worn actigraphs in three experimental protocols. **Study 1** involved healthy young adults with no history of mental illness, in Melbourne, Australia. Their potential risk of future BD was assessed using the well-validated General Behavior Inventory (GBI) [13], [14]. We divided subjects by highest versus lowest deciles of resulting GBI scores into those with apparently *low risk* (*n* = 35; 77.1% women; mean±SD, age  = 20.9±2.3) versus *high risk* for BD (*n* = 35; 65.7% women; age 22.3±3.0 years). Subjects wore an actigraph continuously on the nondominant wrist for seven days. For **Study 2**, in Australia, we recruited currently clinically euthymic, medicated patients diagnosed with type-I BD by DSM-IV criteria (*n* = 15; 46.7% women; age 46.8±12.4) and healthy controls with no history or current clinical evidence of mental illness, low GBI scores, and matched approximately for sex and age (*n* = 15; 46.7% women; age  = 46.7±14.1), who also wore an actigraph for seven days. In **Study 3**, at the University of Parma, Italy, patients with a DSM-IV diagnosis of type-I BD (*n* = 51; 70.6% women; age 43.8±10.5 years) were actigraphically monitored prospectively for 3 days during acute manic (*n* = 21) or mixed manic-depressive states (*n* = 13), episodes of major depression (*n* = 13), and during full clinical recovery (n = 46) sustained for at least 60 days (total of 90 observations).

Measures of output variables representing underlying dynamics of a biological system can exhibit complex fluctuations across different time scales. Based on nonlinear system theory, the multi-scale features of these measures can be characterized [4], [5], [15]. The past decade has seen growing interest in nonlinear dynamical analysis of physiological data [16], [17], and a range of computational approaches have been used to seek hidden signals of pathology from the measures of the time series [18], [19], [20]. In the present study, we focused on *amplitude* behavior since amplitude of motility data is physiologically grounded as a measure of strength of physiological oscillations regulated by the SCN [21], [22].

## Results

### Probability distribution of multi-scale amplitudes

To understand the multi-scale characteristics of amplitude measures of motility data collected with actigraphy, we considered data from Study 1. A wavelet transform[23] (see Methods) was used to extract amplitude of motility rhythms at different time scales ranging from 0.2 to 26 h. Motility data are highly non-stationary due to various extrinsic and intrinsic factors [24], and wavelet transform is a powerful method for obtaining amplitude at multiple time scales from such non-stationary biological signals [15].

**Figure 1:**
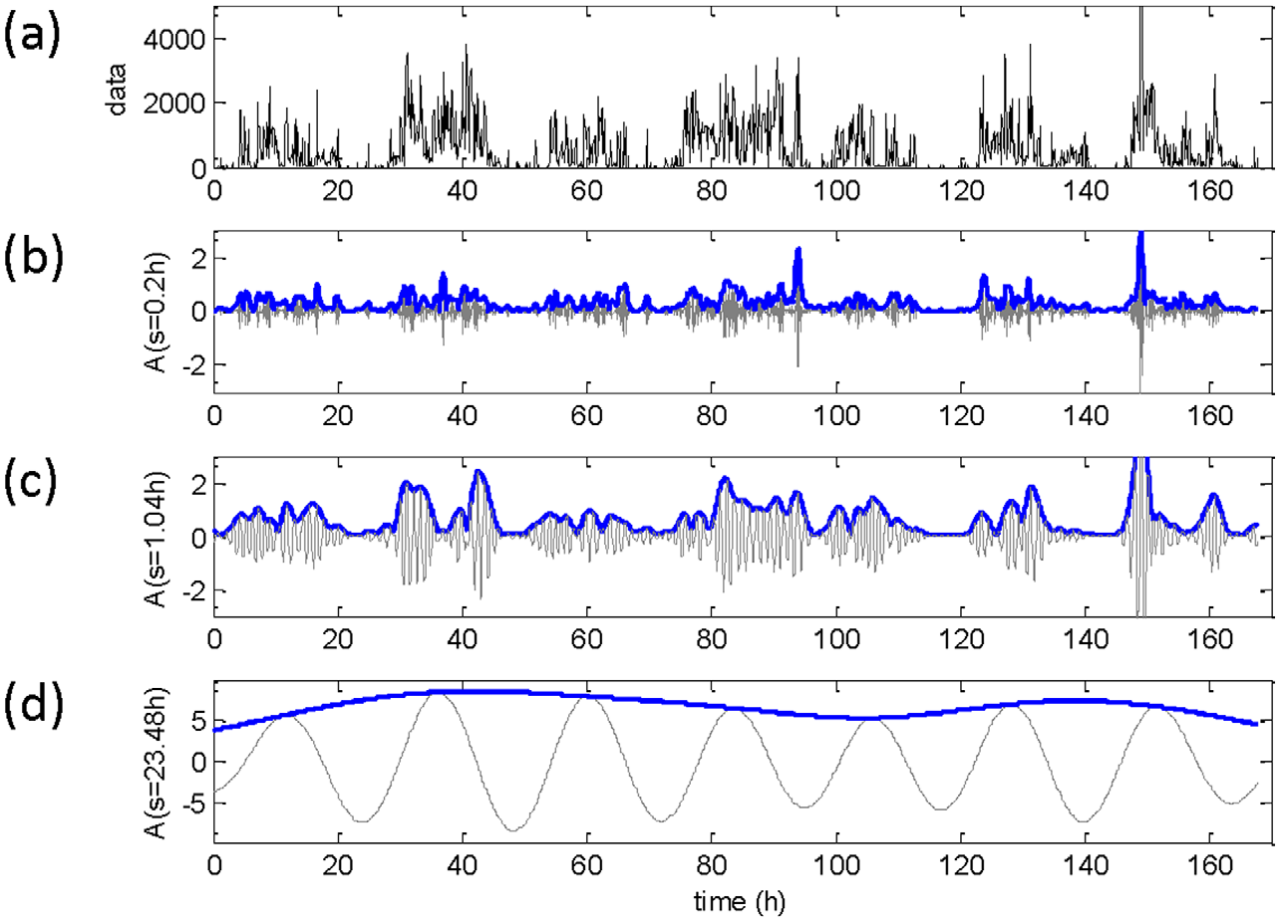
Estimated multi-scale rhythms of motility data obtained by wavelet analysis. **(a)** Raw data in arbitrary units (a.u) along with the rhythms detected at three different time scales. **(b)** The detected rhythm (grey line) along with its amplitude (blue line) for a scale of 0.2 h. **(c)** The corresponding rhythm and amplitude for a scale of 1.04 h. **(d)** The same measure at a scale of 23.48 h. Wavelet amplitudes are in normalized arbitrary units.

**Figure 2:**
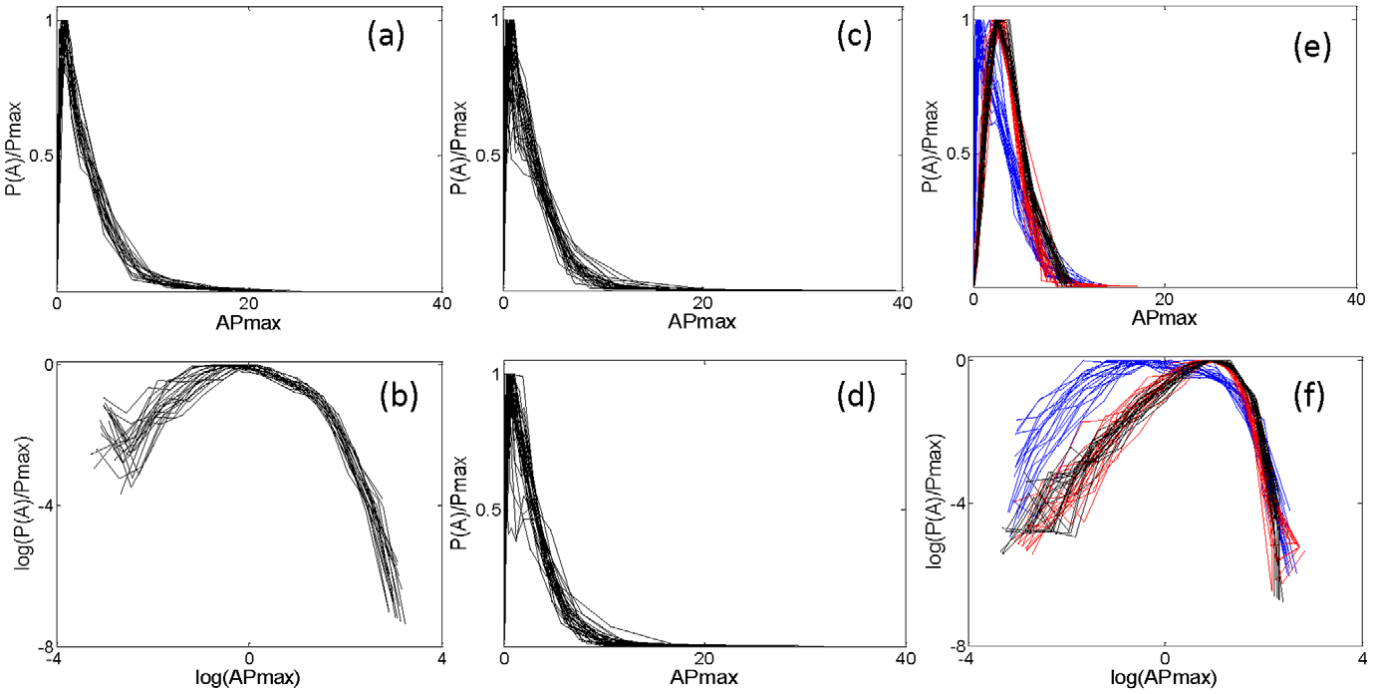
Probability distribution *P(A)* of amplitude, *A*, obtained by wavelet analysis of motility data. (**a**) Rescaled distributions, normalized to provide unit area by rescaling using *P_max_*, of amplitude at a range of time-scales up to 2 h from a subject considered to be at low risk for BD by GBI criteria. (**b**) The same data, log-transformed and showing a long-tail, which share most of the values of log (*AP_max_*). (**c**) Amplitude distribution at a specific scale (s = 0.54 h) for 35 subjects considered to be at low risk for BD by GBI criteria. (**d**) The same for 35 other subjects at high risk for BD. (**e**) Amplitude distribution up to 2 h for a subject at high risk for BD (blue line) and the corresponding surrogate data (red line); the amplitude distribution (black line) obtained from the wavelet analysis of data derived from a Gaussian distribution follows the amplitude distribution of surrogate data. (**f**) Log-transformed amplitude from panel *e*; here, wavelet amplitudes of Gaussian as well as surrogate data have identical distributions without a long-tail, and differ from the distribution of the original data.

Rhythms were observed at circadian (∼24 h) as well as other temporal ranges (minutes or hours). Figure 1 represents an example of motility data along with the multi-scale rhythms obtained using wavelet analysis. The amplitude of rhythms at shorter time scales appeared to be random; to check whether such fluctuation was simply due to noise in the data, we plotted the distribution of amplitudes at a range of time-scales. The distribution of amplitudes obtained at very short time-scales (≤2.0 h) had an apparent long-tail and was nearly collapsed (Figure 2 (a–d)). Such a long-tail distribution is characteristic of nonlinear complex systems near critical points, and the collapse of amplitude distribution represents the scale-invariant feature of such systems [25].

To confirm that the observed long-tail distribution was an intrinsic nonlinear property of the motility data rather than an artifact of wavelet analysis, we performed surrogate data analysis, a standard test to confirm nonlinearity of the data [26]. Surrogate data were obtained by Fourier transform of the original motility data, preserving the amplitude, randomizing the phases, and then computing inverse Fourier transforms. Since both amplitude and the power spectrum were preserved in the surrogate data, it might be expected that the amplitude distribution for the surrogate and original data would be identical.

However, wavelet analysis indicated that the amplitude distribution for the surrogate data followed a Gaussian distribution without a long tail. In addition, we generated artificial data with values obtained from a Gaussian distribution, and repeated the wavelet analysis. The amplitude distribution of such artificial data at lower time scales had a similar distribution to that of the surrogate data (Figure 2 (e–f)). These observations imply the presence of nonlinear interactions in the original motility data and that the long-tail distribution of amplitude was an intrinsic property.

### Derivation of Vulnerability Index (VI) of BD

To characterize the long-tail distribution, we sought the distribution function that best fit the observed amplitude distribution. Although Gamma functions are considered optimal for characterizing such long-tail distributions [27], we compared the goodness-of-fit of the amplitude distribution for both Gamma and Rayleigh [28] distributions. Theoretically, based on the central limit theorem, amplitude at different time-scales for data derived from a Gaussian distribution should follow a Rayleigh distribution, whereas a long-tail distribution characterized by a Gamma distribution should reveal the presence of nonlinear interactions [15]. We found that for scales up to 2.0 h, the fit of the Gamma distribution was better than the Rayleigh distribution based on Akaike Information Criteria (AIC) [29].

**Figure 3:**
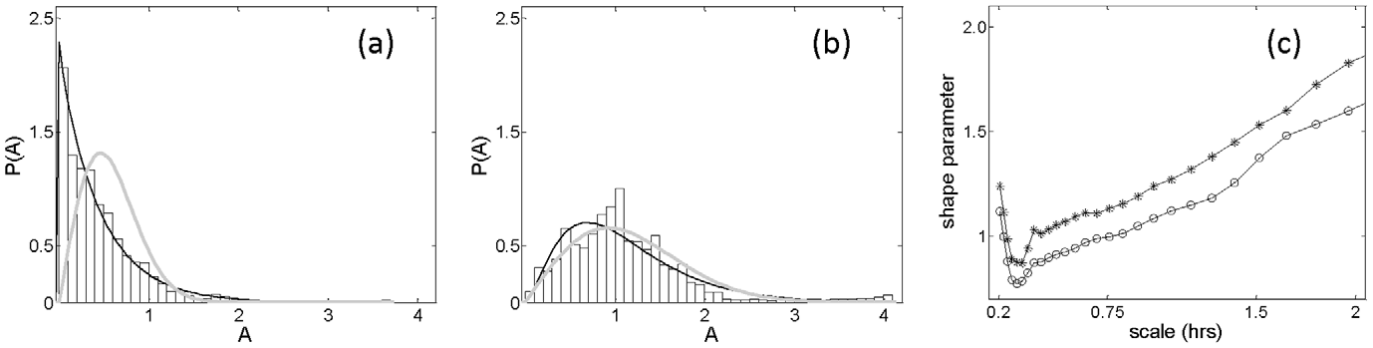
Characterization of the probability distribution of amplitudes using Gamma and Rayleigh functions. (**a**) Probability distribution *P(A)* for a subject considered to be a low risk for BD at a scale s∼0.59 h, along with Gamma (black line) and Rayleigh (grey line) best-fit functions: based on AIC, the Gamma distribution yields a better fit. (**b**) Probability distribution *P(A)* at a scale of s∼3.0 h for the same subject: the fit of the Rayleigh distribution function (gray line) is superior to the Gamma function at this scale, by AIC criteria. (**c**) Average value of the shape parameter, 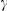, at different scales (hours) for 35 subjects considered to be low risk for BD by GBI criteria (open circles) and another 35 at high risk (filled circles).

To look for any scale-invariant feature in the amplitude distribution at multiple time scales, we plotted the estimated shape parameter, γ, of the Gamma distribution fit at different time scales. We found that the shape parameter exhibited multi-scale behavior (Figure 3). If the shape parameter of the individual distribution showed a constant value for time scales up to 2.0 h, this would indicate scale-invariance. However, the shape parameter decreased at short time scales and gradually increased at longer time scales. Although the long-tail distribution was a property of the data, the amplitude distribution lacked a scale-invariant feature. Since such a non-collapsed nature of the distribution may represent an abnormality of the system, we investigated plausible correlates of this multi-scale behavior to the illness of BD. To quantify the observed behavior, we integrated the shape parameter up to 2.0 h time scales for each subject as 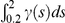. The derived integral, termed a *vulnerability index* (VI), was used in hypothesis-testing analyses.

### VI as a marker for detecting trait of BD

Hypothesis 1 was tested with data from Study 1, in which the *low risk* group, based on lowest decile GBI ratings, had 12% lower mean VI scores (2.11±0.45) than the *high risk* group (2.39±0.42). Logistic regression modeling indicated that VI was significantly associated with presumptive high risk for BD (*F* [*df* = 1; 66] = 8.69, *p* = 0.004), but that sex and age were not. Similarly, testing of Hypothesis 2 with data from Study 2 found 17% higher VI scores among patients diagnosed with BD (2.53±0.49) than in healthy control subjects (2.17±0.40), and that VI score was associated with the diagnosis of BD, based on logistic regression modelling (*F*[*df* = 1;26] = 4.65, *p* = 0.04).

**Figure 4:**
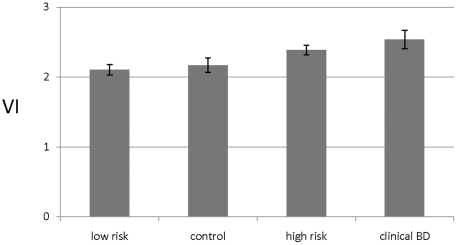
Estimated means (±SEM) computed *vulnerability index* (VI) values in four groups of human subjects of increasing risk or presence of BD. Low risk by GBI criteria (n = 35), healthy controls (n = 15), high-risk by GBI criteria (n = 35), and patients diagnosed with DSM-IV type-I BD who are currently clinically stable or euthymic (n = 15). Curve-fitting found that mean VI increased linearly across these groups (*F* [df = 3; 94] = 4.28, *p* = 0.007, controlling for sex and age).

When all subjects from Studies 1 and 2 were included in a multivariate linear regression modelling, with GBI score as the dependent measure, and controlling for sex and age, the VI score was significantly and independently associated with either high risk for, or diagnosis of BD, and was the only factor associated (*F* [*df* = 3; 96] = 4.94, *p* = 0.001). Likewise, we found a significant linear trend in the mean magnitude of VI measures, ranking: low-GBI score < healthy controls ≤ non-BD patients with high GBI scores < BD patients (Figure 4), with control for sex and age (*F* [*df* = 3; 94] = 4.28, *p* = 0.007, *β* = 0.32, *p* = 0.01).

These findings indicate the presence of a consistent and significant association between VI scores and GBI scores as an indication of trait for BD in healthy subjects with high versus low GBI scores, and in comparison of BD patients with sex- and age-matched healthy controls.

### Circadian amplitude and detrended fluctuation measure of motility data

**Figure 5:**
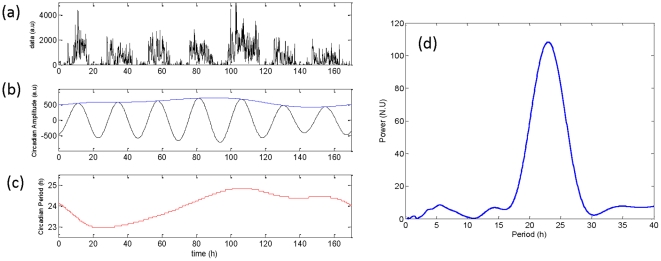
Estimated predominant component of motility data. **(a)** The raw data in arbitrary units (a.u). **(b)** The estimated circadian rhythm (black line) with its amplitude (blue line). **(c)** The estimated circadian period (red line) in hours. Due to the non-stationary nature of the data, the estimated amplitude and the period show cycle-to-cycle variability. **(d)** Normalized scalogram at a specific time of 32 hr. The peak power occurred at a period of 23 h

To check whether vulnerability to BD would typically be inferred from more common analyses of ∼24-hour (circadian) features of actigraphic data, we estimated the predominant component using wavelet transform along with a ridge extraction algorithm [30]. Figure 5 represents an example of the estimated predominant amplitude and the period from a representative human subject. The predominant component of motility data was in the circadian range. However the predominant amplitude in arbitrary units (mean ± SD ranked for *low risk*: 446±150, versus *high risk*: 411±145 subjects, BD patients: 332±132, versus healthy controls: 435±146) and the predominant period in hours (mean ± SD ranked for *low risk* subjects: 24.0±0.20, *high risk* subjects: 23.9±0.3, BD patients: 24.0±0.05, healthy controls: 24.0±0.1) of the circadian component did not show significant differences among the subject-groups.

Detrended fluctuation analysis has indicated that motility data have long range correlations with a scale-invariant feature in temporal fluctuations ranging up to 24 h, and that these correlations can distinguish elderly persons diagnosed with Alzheimer's disease from healthy elderly controls[31]. By application of detrended fluctuation analysis [31] of the present motility data, we considered whether such fluctuations were different among the various groups of test subjects. Although we found a scale-invariant feature in temporal fluctuations, its characterizing parameter, scaling exponent, (mean ± SD) did not differ significantly among subject-groups. This measure ranked: *low risk* subjects, 0.98±0.07 = *high risk* subjects, 0.98±0.06> healthy controls, 0.96±0.05> BD patients, 0.94±0.09.

### VI as a marker for detecting different morbid phases of BD

We used data from Study 3 to test whether differences in VI values were associated with varying clinical states of BD (Hypothesis 3). Statistical findings are shown based on linear correlations (Table 1), which were supported by nonparametric Spearman rank-correlations (not shown). Regression modelling, with control for sex and age indicated that VI was significantly and uniquely associated with both self- (*β* = 0.31, *t* = 2.76) and clinician-rated mania (*β* = 0.33, *t*  = 3.07, both *p* = 0.01), whereas age was a lesser covariate (*β = *0.239, *p = *0.05). The pattern of findings was replicated with multi-level modelling to account for nesting of assessments within subjects: and a significant relationship was found between VI and both self- (*t* [*df* = 78] = 2.97, *p* = 0.004) and clinician-rated mania (*t* [*df* = 81] = 2.54, *p* = 0.013).

**Table 1:** Correlations of individual values of vulnerability index (VI) with self- and clinician-ratings of manic and depressive symptom-severity.

Correlate	*r*-value	*p*-value
***Manic Symptoms***		
Clinician-rated with YMRS	+0.304	0.005
Self-rated	+0.305	0.006
***Depressive Symptoms***		
Clinician-rated with HDRS)	−0.017	0.877
Self-rated)	−0.114	0.313

**Figure 6:**
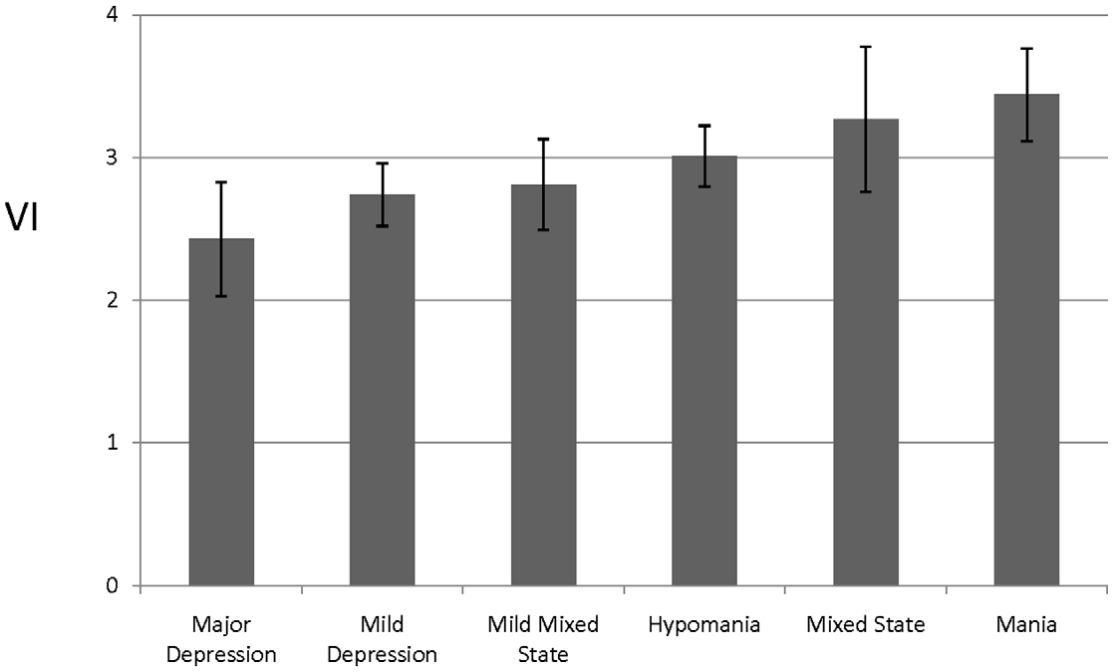
Mean (±SEM) computed *vulnerability index* (VI) values associated with various mood states of patients meeting DSM-IV diagnostic criteria for type-I BD. VI rises consistently and significantly with increasing manic features, from major depression, through mild depression or dysthymia, subclinical or mild mixed manic-depression, hypomania, mixed major manic-depressive states, to current mania-all in BD patients.

Importantly, regression analyses found no correlation of VI with depressive symptom ratings (Hamilton Depression Rating Scale [HDRS] score or self-rated depression *F* [*df* = 3;79] = 2.32, and *F* [*df* = 3;76] = 1.21 both p>0.05). However, there was a significant increase in mean VI values across mood-states, ranking: major depression < minor depression < euthymia < minor mixed-states < hypomania < major mixed-states < mania (Figure 6).

## Discussion

We identified a novel scaling behaviour of the amplitude measures of locomotor activity in human subjects. Importantly, an index of this multi-scale behaviour, the “vulnerability index” (VI), is found to be clinically meaningful. That is, higher VI scores were found in subjects with apparently high versus low vulnerability to BD (high versus low GBI scores), and in BD patients versus healthy, matched controls. We propose that an elevated value of VI can serve as a novel and effective indicator of trait for BD as well as being characteristic of subjects with clinically diagnosed BD. In these subject-groups vulnerability for BD manifested in the amplitude characteristics of activity data at lower time scales, which appeared as noise to the naked eye, and were unrelated to the temporal long-range correlations or typically-analysed circadian features of motility rhythms.

The VI is specifically sensitive to mania and manic symptoms, which distinguish BD from other mood disorders. However, VI is insensitive to depression, consistent with the view that mania and depression in BD have dissimilar pathophysiologies [32]. Furthermore, the increasing variations of VI across different mood states, as shown in Figure 6, suggest that the VI might be a specific and sensitive indicator of a gradually emerging component of psychomotor activation from predominantly inhibited or psychomotorically retarded depressive states towards mixed or manic states.

The VI may reflect SCN function, which has been implicated in the pathophysiology of BD [33]. Of various SCN-related rhythms, human motility can easily be monitored noninvasively and inexpensively, either intermittently for short times, or continuously for long periods under naturalistic conditions, making it particularly well-suited to longitudinal tracking of illness-course and treatment responses in BD patients. Further research is warranted to investigate the biological substrate of VI and to confirm its potential utility as an objective marker of risk for, or presence of BD, and of its selectivity for manic versus depressive features. It might also bear testing as early indicator of impending clinical relapse into manic or mixed states of BD arising from depression or euthymia.

## Methods

### Ethics Statement

All subjects provided written informed consent for aggregate and anonymous reporting of data arising from their clinical and actigraphic assessments. Study 1 was approved by Swinburne University Human Research Ethics Committee; Study 2 was approved by Bendigo Health Care Group Ethics Committee and Study 3 was approved by the Ethics Committee of the University of Parma Medical Center, in full accordance with international standards for the ethical use of human subjects in research.

### Psychiatric measures

The 73-item GBI is a reliable and validated self-report measure with stable between-subject differences that are reported to reflect vulnerability to BD. [34], [35]. In Study 3, clinician ratings of symptom-status were made with the Young Mania Rating Scale (YMRS) [36] and the Hamilton Rating Scale for Depression (HDRS) [37]. Subjects in Study 3 also self-rated symptoms on a series of 10-cm visual analog scales (29 items) based on items of the YMRS and HDRS as well as the Bonn Scale for the Assessment of Basic Symptoms (BSABS) [38].

### Subjects

**Study 1.** Exclusion criteria included past history of mania/hypomania, working shift work, or having a physical condition that would confound activity measurement. The highest versus lowest deciles of the distribution of GBI scores, identified 35 subjects considered to be at high-risk, and 35 at low-risk for BD from an initial sample of 358. Mean GBI score differed by more than ten-fold between subjects with low versus high apparent risk for BD (10.5±6.20 vs. 109±26.3, *F* [*df* = 1; 68]) = 465, *p*<0.001). **Study 2**: Clinical subjects were recruited from an outpatient public mental health service in Victoria, Australia, who met DSM-IV criteria for a diagnosis of type-I BD as determined by an experienced research psychiatrist. Sex- and age-matched healthy controls were recruited through network of researchers. **Study 3**: Patients were recruited as inpatients at the Section of Psychiatry of the University of Parma, Italy, whose DSM-IV diagnosis of type I BD was supported by semi-structured interviews by a research psychiatrist over prolonged (7.43±2.24 years) follow-up as outpatients.

### Activity measurement

Actigraphy is commonly used to document rest/activity cycles under naturalistic conditions and to characterize abnormal sleep/wake behaviour and altered circadian rhythms amongst ill or euthymic BD patients. In the present studies, subjects wore an actigraph continuously on the non-dominant wrist under naturalistic conditions for 3–7 days. The Mini-Mitter Actiwatch® 64 (Respironics, Inc. Bend, OR, USA) was used in Studies 1 and 2, and AMA-128K Mini-Motionlogger® Actigraph (Ambulatory Monitoring, Inc. [AMI], Ardsley, NY, USA) in Study 3. Activity data were obtained at 32 Hz and integrated at every 0.1 h.

### Data Analysis

The continuous wavelet transform of digitally acquired motility data, 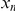 with 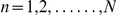 is obtained by the convolution of the data with a scaled and translated version of a mother wavelet [23], as:
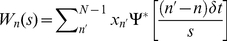
(1)where 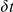 = 0.1 hr, 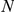 is the total number of data points, 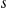 is the scale defined in a dyadic representation as 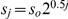 with 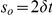 and 4 sub-octave per octave of the dyadic scale to obtain a total number of 57 scales with 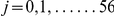. 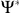 represents the complex conjugate of the normalized wavelet function, where:
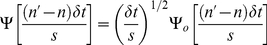
(2)


We performed this convolution with a Morlet wavelet, a plane wave modulated by a Gaussian function defined as
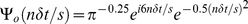
(3)Since the Morlet wavelet is a complex function, the obtained wavelet transformation of the data, 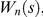 is also complex with a real part and an imaginary part. Therefore, corresponding to each scale the amplitude is defined as 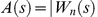. The obtained amplitudes are in normalized units.

To determine the predominant component of the motility data we employed the wavelet transform with an increased resolution of scale with 256 sub-octaves per octave in the dyadic representation. At each time point, using a ridge extraction algorithm[30], the scale at which the normalized scalogram has a peak value is detected. The corresponding amplitude multiplied by the appropriate de-noising factor [23] at this scale is the predominant amplitude and the corresponding period is the predominant period. Our approach for determining the predominant component is similar to the method employed for estimating circadian oscillations from bioluminescence data [39], [40].
